# Time evolution of frontal plane dynamic balance during locomotor transitions of altered anticipation and complexity

**DOI:** 10.1186/s12984-020-00731-0

**Published:** 2020-07-18

**Authors:** Wentao Li, Nathaniel T. Pickle, Nicholas P. Fey

**Affiliations:** 1 Department of Mechanical Engineering, The University of Texas at Austin, 204 E Dean Keeton Street, Austin, TX 78712 USA; 2 Biomedical and Life Science Division, CFD Research Corp, Huntsville, AL USA; 3 Department of Physical Medicine and Rehabilitation, The University of Texas Southwestern Medical Center, Dallas, TX USA

**Keywords:** Biomechanics, Gait, Walking transitions, Dynamic balance, Cognition

## Abstract

**Background:**

Locomotor transitions between different ambulatory tasks are essential activities of daily life. During these transitions, biomechanics are affected by various factors such as anticipation, movement direction, and task complexity. These factors are thought to influence the neuromotor regulation of dynamic balance, which can be quantified using whole-body angular momentum (*H*). However, the specific effects of these factors on balance during transitions are not well understood. The ability to regulate dynamic balance in the presence of these contextual factors is especially important in the frontal plane, as it is usually challenging to maintain walking balance in the frontal plane for individuals with neuromuscular impairments. The purpose of this study was to apportion their effects on the time evolution of frontal plane dynamic balance during locomotor transitions of healthy, unimpaired individuals.

**Methods:**

Five healthy young subjects performed 10 separate types of transitions with discrete combinations of factors including complexity (straight walking, cuts, combined cut/stair ascent), cut style (crossover, sidestep), and anticipation (anticipated and unanticipated). A three-way analysis of variance (ANOVA) was used to compare the maxima, minima, and average rates of change of frontal-plane *H* among all transitions.

**Results:**

Before transition, within anticipated state peak value of *H* increased 307% in crossover style relative to sidestep style (*p* < 0.0001). During Transition Phase, within unanticipated state the magnitudes of average rate of change and peak value increased 70 and 46% in sidestep style compared to crossover style (*p* < 0.0001 and *p* = 0.0003). Within sidestep style, they increased in unanticipated state relative to anticipated state. Later in Correction Phase, within both anticipation states peak value of *H* increased 41 and 75% in cut/stairs transitions relative to cuts (*p* = 0.010 and *p* < 0.0001). For cut/stairs transitions, peak value of *H* increased 45% in unanticipated state compared to anticipated state (*p* = 0.0001).

**Conclusions:**

These results underlined the detrimental effects of unanticipated state and task complexity on dynamic balance during walking transitions. These findings imply increased demand of neuromuscular system and functional deficits of individuals with neuromuscular disorders during these tasks. In addition, cutting style influenced frontal plane dynamic balance before transition and in response to unanticipated direction change. Collectively, these results may help identify impaired balance control of fall-prone individuals and inform interventions targeting specific destabilizing scenarios.

## Background

Humans are frequently required to make cognitive decisions and respond to external stimuli during walking in uncontrolled “real world” environments. These cognitive factors can lead to locomotor transitions that may involve cuts (changing direction), moving from level to uneven terrain, or complex combinations of these tasks. The ability to perform these transitions is crucial for activities of daily living, as cuts alone compose up to 50% of everyday movements [1]. Cutting movements are well-researched in the context of sports movements involving jogging or running [2–5]. However, much of the literature regarding slower-paced walking tasks have focused on steady-state walking [6–9], and thus locomotor transitions are not as well understood in the context of typical daily activities.

Steady-state walking exhibits “orbital stability”, with each step deviating only slightly from the kinematics of the previous step [10]. In contrast, transitions between locomotion modes are not periodic, and balance must be maintained while responding to entirely new task demands. There are two distinct styles of cutting movements, each with its own distinct medial/lateral biomechanics during walking [11] and running [12, 13]: a crossover (rotating the trailing, swing leg toward the leading, implanted leg) or sidestep (rotating the trailing, swing leg away from the leading, implanted leg). Among several stepping maneuvers related to these two styles, young and old individuals were more likely to select a sidestep maneuver to maintain balance in response to walk-in-place lateral perturbations [14]. While a sport-like agility test indicated the similar preference of sidestep style, researchers also claimed that sidestep maneuver may place greater injury risk on the young female athlete relative to crossover style [13]. Although different cut styles were related to balance regulation during walk-in-place task [14], it is unclear how healthy adults maintain dynamic balance with each maneuver during walking cut transitions. Most of previous research on walking cuts primarily targeted at understanding knee injury mechanisms [15, 16] and joint kinetics [17], rather than investigating dynamic balance. Moreover, locomotor transitions may be of movement complexities that could further affect human biomechanics during walking. For instance, joint power generation was increased in young adults during transitions of increased complexity involving both level changes and obstacle avoidance [18]. Transitions from level ground to stair ascent require larger hip and knee joint moments relative to level walking and thus are biomechanically challenging [19, 20]. Combined transitions involving both a cut and switching from level walking to stair ascent are likely even more challenging, but this type of complex transition has not been thoroughly investigated.

Locomotor transitions can also be influenced by anticipation, which is a cognitive rather than environment factor. During locomotion, the nervous system maintains an “internal model” of the dynamics of the body, and uses this model in a feedforward sense to coordinate neural control of movement in preparation for anticipated motions [21, 22]. Unanticipated tasks interrupt this locomotor planning and can therefore be challenging, especially for individuals with deficits in feedback neuromuscular control, such as impaired proprioception [23]. For example, the biomechanics of unanticipated cuts may lead to knee loading mechanics that increase risk of knee ligament injury [15]. In response to unanticipated walk-in-place lateral perturbations, the young and elderly implemented different stepping maneuvers to maintain balance but suffered from high frequent collisions of limbs during stepping [14]. However, the majority of studies that have investigated anticipatory adjustments during locomotor transitions have focused primarily on joint mechanics. For example, anticipatory changes of center-of-mass kinematics, joint angles, and EMG were found before transitioning from level-ground walking to stair ascent [24]. It is not well investigated how dynamic balance is modified during unanticipated locomotor transitions. Thus, it remains unclear how the specific contextual factors of cut style, task complexity and anticipation affect regulation of dynamic balance during locomotor transitions.

One metric for assessing dynamic balance during walking is whole-body angular momentum (*H*), which is tightly regulated by unimpaired individuals during level-ground walking [8]. Regulation of *H* is achieved primarily through muscle force generation [25, 26]. *H* is also a valuable quantity to study balance because it relates to the net external moment (*M*_*external*_) about the body center of mass (COM) by the equation $\dot{H}=\sum {M}_{external}$. The external moment on body is the cross product of the external moment arm and the ground reaction force (GRF). Thus, *H* is directly related to the human whole-body dynamics and is not as reliant on simplifying kinetic assumptions of inverted pendulum model as other commonly used measures of dynamic balance, such as margin of stability [27].

Frontal-plane *H* in particular is useful for identifying altered dynamic balance control in individuals with a variety of neuromuscular impairments. For example, in individuals post-stroke the magnitude of change in frontal-plane *H* during stance of the paretic leg is correlated with lower (worse) Dynamic Gait Index and Berg Balance Scale scores [28]. Although the elderly may have unique strategies performing movement tasks [29, 30], elderly individuals with vestibular balance impairment also have increased frontal-plane *H* during gait compared to age-matched peers without vestibular dysfunction [31]. The range of *H* in people with unilateral transtibial amputation is larger during prosthetic leg stance compared to able-bodied subjects across several different walking speeds [32]. During stair ascent, the range of frontal-plane *H* is greater compared to level-ground walking in able-bodied individuals, and is associated with altered GRFs and external moment arms during stair ascent walking compared to level walking [33]. The unique demands of stair ascent may be particularly challenging for individuals with neuromuscular impairments. For example, people with transtibial amputation also have a greater range of frontal-plane *H* during stair-ascent compared to level-ground walking [34]. However, this increased range of *H* and the associated changes in GRFs and external moment arms may be more difficult to achieve due to reduced proprioception and control in the prosthesis compared to a biological leg. Furthermore, the transition from level-ground walking to stair ascent may be more challenging than steady-state stair ascent. Thus, it is important to understand regulation of dynamic balance during complex transitions that may pose a risk to people with neuromuscular impairments. However, dynamic balance (i.e., regulation of *H*) before *and* during these complex and challenging transitions, particularly when they are unanticipated, is not yet well understood in unimpaired individuals.

Therefore, the purpose of this study was to apportion the effects of task anticipation, cutting style, and complexity on the time evolution of (i.e. time-varying) frontal-plane dynamic balance during locomotor transitions of young, healthy, unimpaired individuals based on *H*. We hypothesized that the peak values of frontal-plane *H* would be larger during unanticipated transitions of increased complexity (i.e., combined cut/stair-ascent). We expected this because steady-state stair ascent has a larger range of *H* compared to level-ground walking, and we expected the interruption of neural planning during an unanticipated transition to stairs to further increase the peak values of *H*. We also hypothesized that able-bodied individuals would have higher average rate of change of frontal-plane *H* during unanticipated transitions of increased complexity. The average rate of change of *H* is equal to the average net external moment about the body COM, and thus correcting for errors in unanticipated transitions was expected to increase the required net external moment in the frontal plane.

## Methods

### Subjects and protocol

**Fig. 1:**
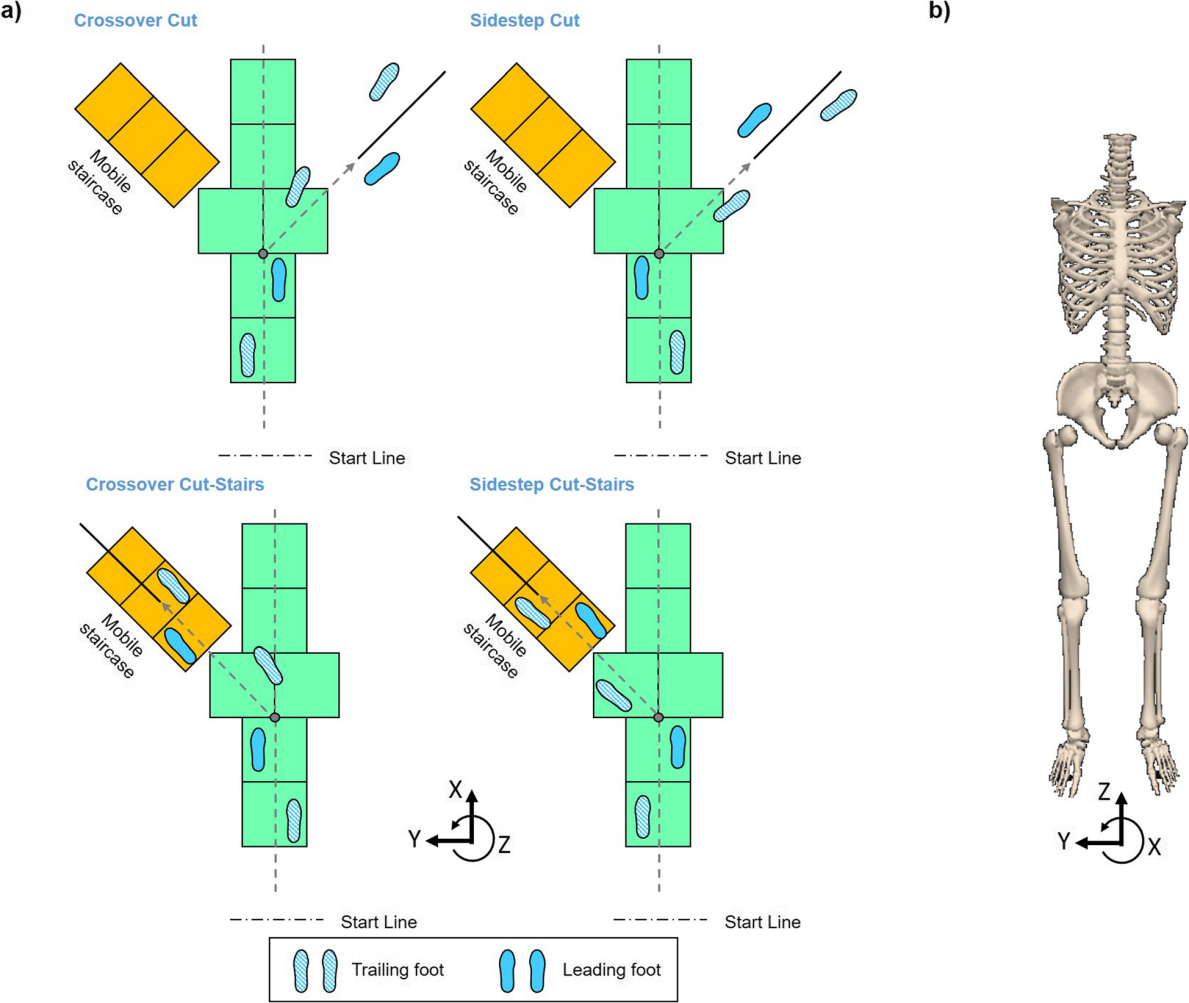
**a** Diagram depicting experiment setup and a subject preforming crossover cut (left top) and sidestep cut to stair ascent transitions (right bottom) using the left (trailing) leg, sidestep cut (right top) and crossover cut to stair ascent transitions (left bottom) using the right (trailing) leg. Auditory cue in unanticipated transitions was given at the initiation of single-leg support of the leading leg (first trailing leg toe-off). Grey dot on the walkway indicates the transition point. **b** Diagram depicting the 8-segment human body model and the direction of frontal-plane angular momentum

Five young healthy unimpaired individuals (4 females, 1 male) with an average age of 27.7 (SD = 2.8) years, mass of 52.6 (SD = 2.8) kilograms and height of 1.68 (SD = 0.06) meters participated in this study. All participants were free of any known history of neurological or orthopedic disorders or lower extremity injury prior to the participation in this study. All participants provided written informed consent to participate in the experimental protocol that was approved by the Institutional Review Board. The lab setup consisted of an over-ground straight-line walkway, a level-ground cutting (45°) direction to the right, and a mobile staircase at 45° to the left for combined cut/stair-ascent (Fig. 1a). Each participant performed straight-line walking, crossover cut, sidestep cut, crossover cut/stair-ascent, and sidestep cut/stair-ascent (Fig. 1b) under anticipated and unanticipated conditions. First, each participant completed 10 baseline straight walking trials, followed by 20 anticipated cut trials in a block (5 of each style and complexity) randomized order. Subsequently, each participant performed 30 unanticipated trials in a fully randomized order, including 10 unanticipated straight walking and 20 unanticipated transition trials (5 of each style and complexity). Short breaks were provided between sessions. In baseline straight walking, participants were asked to start with their preferred legs and walk in their normal walking speeds. In anticipated cut trials, each subject was asked to start with their left leg for the first 2 blocks and right leg for the last 2 blocks. Participants were asked to “walk-cut” or “walk-stair” for each block subsequently. In unanticipated trials, subjects were asked to start with their left leg for the first block (15 trials) and right leg for the second block. A randomized auditory cue of “stair”, “cut”, or “walk” was given at the initiation of single-leg support of the leading leg (i.e., the toe-off of the trailing leg), approximately one-half step preceding a visible transition point (Fig. 1). The number of each type of auditory cue was controlled to be equal (5 ± 1 each). The start points were at least 2 steps away from the transition point.

### Data collection and processing

A 10-camera motion capture system (Motion Lab Systems, Baton Rouge, LA, USA) operating at 120 Hz was used to track 42 reflective markers placed bilaterally on the trunk, pelvis, thighs, shanks and feet. An 8-segment model of each subject (torso, pelvis, thighs, shanks, and feet) was constructed based on a static trial. Biomechanical data were processed with Visual3D (C-Motion, Germantown, MD, USA), and 3D motion capture data were filtered using a low-pass Butterworth filter with cut-off frequency of 6 Hz.

*H* about the whole-body center-of-mass was calculated in Visual3D as

$$ \overset{\rightharpoonup }{H}={\sum}_{i=1}^8\left[{I}_i\overset{\rightharpoonup }{\omega }+\left({\overset{\rightharpoonup }{r}}_i-{\overset{\rightharpoonup }{r}}_{COM}\right)\times {m}_i\left({\overset{\rightharpoonup }{v}}_i-{\overset{\rightharpoonup }{v}}_{COM}\right)\right] $$

where *I*_*i*_, ${\overset{\rightharpoonup }{\omega}}_i$ are the moment of inertia tensor and angular velocity, respectively, of the *i* th segment about the body’s COM in the lab frame; ${\overset{\rightharpoonup }{r}}_i$ and ${\overset{\rightharpoonup }{v}}_i$ are the position and velocity, respectively, of the *i* th segment’s COM in the lab frame; ${\overset{\rightharpoonup }{r}}_{COM}$ and ${\overset{\rightharpoonup }{v}}_{COM}$ are the position and velocity, respectively, of the body’s COM; *m*_*i*_ is the mass of the *i* th segment. *H* was normalized by body mass and height of each subject. The direction of *H* in this study was aligned with the lab reference frame. Positive frontal-plane *H* indicates rotational momentum toward the “leading leg”, while negative *H* indicates momentum away from the “leading leg” (Fig. 1b). We defined the leading leg as the implanted leg (stance leg) during turning transition (Fig. 1a).

We analyzed consecutive maxima (P1, P2) and minima (N1, N2) of frontal-plane *H*, as well as the average rate of change between each maxima and minima (P1-N1, N1-P2, P2-N2) during two consecutive strides, from the first heel strike of the trailing leg to the third heel strike (Fig. 1a). We sub-divided the entire transition movement into four phases (Fig. 1a): Preparatory, Transition, Correction, and Completion that were defined by gait events in each leg. The Preparatory Phase occurred before the cue, starting at the first trailing leg heel strike and ending at toe-off of the trailing leg (occurrence of the cue). The Preparatory Phase encompassed positive peak P1. The Transition Phase began with the cue and lasted throughout leading leg stance, ending with leading leg toe-off. Transition Phase comprised negative peak N1, and the average rate of change between P1-N1. Adjustment for errors in the transition occurred during Correction Phase, which started at leading leg toe off, lasted throughout trailing leg stance, and ended with the next trailing leg toe off. Correction Phase included positive peak P2, as well as the average rates of change between N1-P2. The final phase was Completion Phase, in which the person reached a new surface to finish the transition task. Completion Phase was defined as the final leading leg stance, ending with the final trailing leg heel strike, and comprised negative peak N2 and the average rates of change between P2-N2.

### Statistics

The Shapiro-Wilk test was performed to check the normality assumption of the data. Then to determine the effects of anticipation (anticipated, unanticipated), cutting style (crossover, sidestep), and complexity (cut, combined cut/stair ascent) on dynamic balance during each phase of transition, a three-way analysis of variance (ANOVA) was used to compare the maxima, minima, and average rates of change of frontal-plane *H* among all transitions. When the ANOVA indicated significant main or interaction effects (α = 0.05), post hoc comparisons were performed to test for significant differences using Bonferroni’s correction in MATLAB (The MathWorks Inc., Natick, MA, USA). Partial eta squared (η_p_^2^) was used to calculate effect size for statistically significant results. Small, medium and large effect sizes were indicated by η_p_^2^ values greater than 0.01, 0.06 and 0.14, respectively [35]. We then compared the peak values and average rates of change for anticipated and unanticipated straight-line walk using t-tests (α = 0.05), and no significant difference was found. Finally, we compared the peak values and average rates of change of frontal-plane *H* in transition with straight-line walk using t-tests (α = 0.05).

## Results

**Fig. 2:**
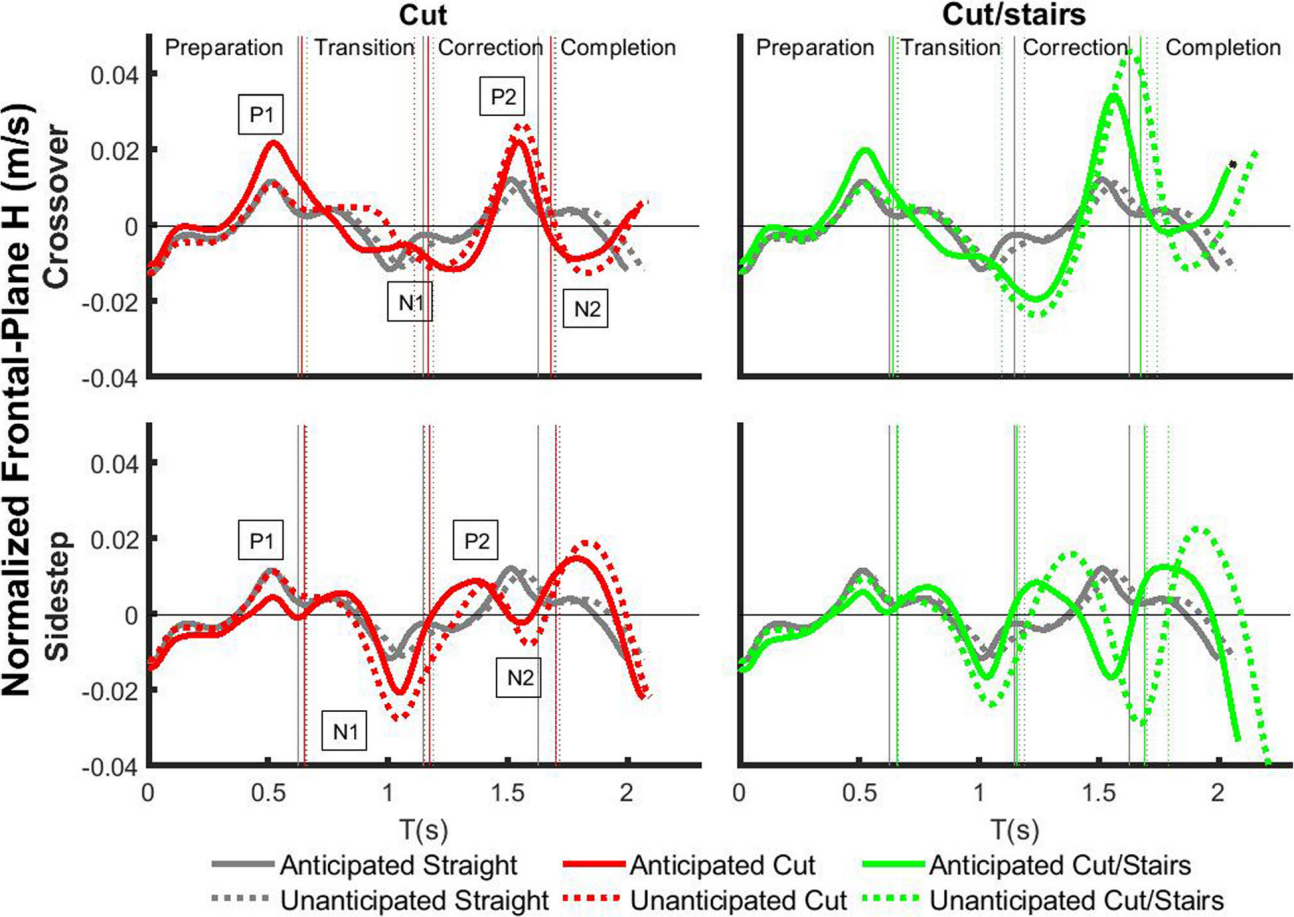
Time-varying frontal-plane group averaged *H* during two consecutive strides of each transition condition*.* Black lines represent for anticipated and unanticipated straight-line walking, while color lines represent for transitions. Solid lines indicate anticipated conditions, while dash lines indicate unanticipated conditions. Vertical lines represent chronological events of the first toe-off of the trailing leg (occurrence of the cue), the first leading leg toe-off, and the second trailing leg toe off

We analyzed the consecutive peak values of frontal-plane *H*, as well as the average rate of change between each peak during two consecutive strides (Fig. 2). We observed time lags of the peak *H* between different cut styles. Positive peak P1 was found at the first leading leg heel-strike for all walking tasks. While negative peak N1 was at the tailing leg heel-strike for sidestep style transitions and straight walk, it was found around the leading leg toe-off for crossover style transitions. Positive peak P2 was around leading leg toe-off for sidestep style transitions, while it was around leading leg heel-strike for crossover style transitions and straight walk. Negative peak N2 was found near leading leg heel-strike for sidestep style transitions, trailing leg toe-off for crossover styles, and trailing leg heel-strike for straight walk.

**Fig. 3:**
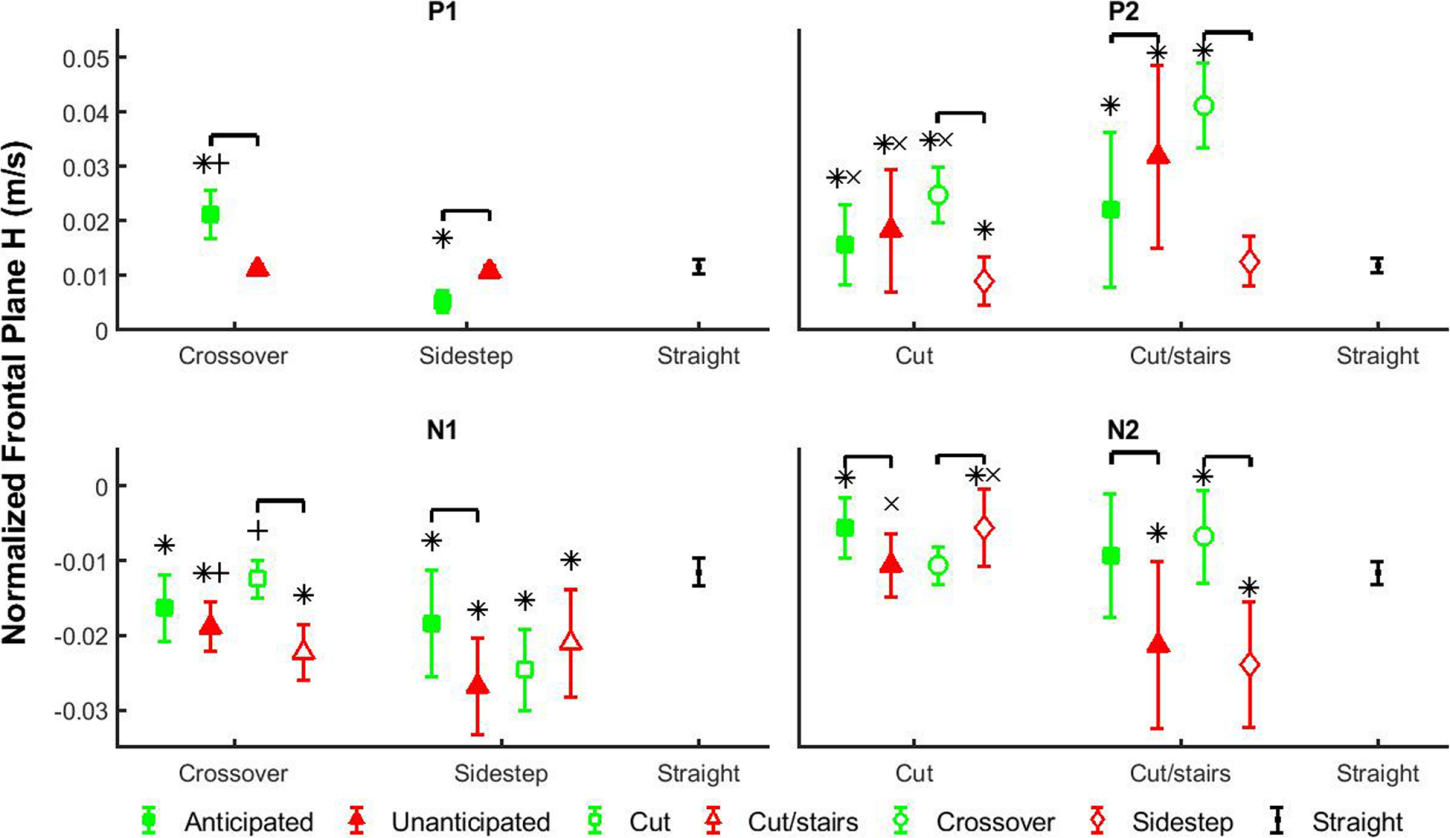
Average and standard deviation of the peak values (P1, N1, P2, N2) of frontal-plane whole-body angular momentum (*H*) in each transition phase. Comparisons were performed among anticipation states (filled shapes), cutting styles (unfilled circles and diamonds) and complexity tasks (unfilled squares and triangles). Green filled squares indicate anticipated states, and red filled triangles indicate unanticipated states. Green unfilled squares indicate cuts, and red unfilled triangles indicate cut/stairs transitions. Green unfilled circles indicate crossover styles, and red diamond indicate sidestep styles. Each marker above represents an interaction scenario of two fixed factors, and consists of different levels of the other fixed factor. Comparisons were also performed between each transition scenario and straight walk. Black dots indicate straight walk in both anticipation states. Brackets indicate significant differences between two transition scenarios. Significant differences between each transition scenario and straight walking are indicated by ‘*’. ‘+’ indicates significant differences between crossover and sidestep cut styles within the same anticipation states, and ‘×’ indicates significant differences between cut and cut/stairs within the same anticipation states or the same cut styles

During the Preparatory Phase, P1 had significant anticipation and cutting style main effects, as well as a significant anticipation by cutting style interaction effect (Table 1). P1 was significantly different in anticipated transitions compared to straight walk, while the unanticipated values were not different relative to straight walk (Fig. 3). Within anticipated state, P1 increased 307% in crossover style relative to sidestep style (*p* < 0.0001; Table 2). Furthermore, P1 was larger in crossover styles, but smaller in sidestep style compared to straight walk.

**Table 1:** The *p*-values from statistical analyses

			PREPARATORY	TRANSITION		CORRECTION		COMPLETION	
			P1	P1-N1	N1	N1-P2	P2	P2-N2	N2
ANOVA main effects									
Anticipation			0.012	0.02	0	–	0*	0*	0*
Cutting Style			0*	0.001	0	0*	0*	0*	0*
Complexity			–	–	0.022	0*	0*	0*	0*
ANOVA interaction effects									
Anticipation * Cutting Style			0*	0*	0.026	–	–	–	–
Anticipation * Complexity			–	–	–	–	0.011	0	0.005
Cutting Style * Complexity			–	0.003	0*	–	0*	–	0*
Anticipation * Cutting Style * Complexity			–	–	–	–	–	–	–
Pairwise comparisons									
Anticipation	Cut Style	Complexity							
Anticipated	X	(X)	0*	–	–		−0.01	(−)	(−)
Unanticipated	X	(X)	–	0*	0		(0*)	(0*)	(0*)
X	Crossover	(X)	0*	−0.037	−0.015		(0*)		(−)
X	Sidestep	(X)	0	0* (−)	0.001 (−)		(−)		(0*)
X	(X)	Cut		0	−0.017		- (0*)	–	0.021 (0.020)
X	(X)	Cut/Stairs		(−)	(−)		0 (0*)	0*	0* (0*)

The *p*-values from statistical analyses of maxima (P1, P2), minima (N1, N2) and average rate of change (P1-N1, N1-P2, P2-N2) of normalized frontal-plane *H* during four phases are shown. Main, interaction effects of ANOVA and pairwise comparison results with *p* < 0.0005 (0.0001) are listed as 0 (0*), and “-” denotes results that were not significant. Pairwise comparisons were only performed when interaction effects were significant, and “X”, “(X)” indicate comparisons between different levels of the associated main factors

**Table 2:** Mean (standard deviation) of *H*

			PREPARATORY	TRANSITION		CORRECTION		COMPLETION	
			P1	P1-N1	N1	N1-P2	P2	P2-N2	N2
Anticipated	Crossover	Cut	22 (5)	−48 (11)	−13 (2)	126 (39)	22 (4)	−120 (30)	−9 (2)
		Cut/Stairs	21 (4)	−57 (10)	−20 (3)	172 (13)	35 (3)	− 131 (29)	−2 (2)
	Sidestep	Cut	5 (2)	−48 (6)	−21 (3)	94 (7)	9 (1)	−59 (17)	−3 (3)
		Cut/Stairs	6 (2)	−44 (8)	−17 (3)	132 (14)	9 (3)	−79 (12)	−17 (3)
Unanticipated	Crossover	Cut	11 (1)	− 33 (5)	−12 (3)	120 (30)	27 (5)	− 153 (26)	−13 (1)
		Cut/Stairs	11 (1)	−50 (6)	−25 (3)	181 (19)	47 (6)	−253 (26)	−12 (5)
	Sidestep	Cut	11 (1)	−77 (9)	−29 (5)	105 (25)	9 (7)	−96 (52)	−9 (5)
		Cut/Stairs	10 (1)	−66 (18)	−25 (8)	129 (23)	16 (1)	−166 (16)	−31 (5)
Anticipated	Straight		12 (1)	−42 (3)	−12 (2)	45 (3)	12 (1)	−47 (7)	−12 (2)
Unanticipated	Straight		11 (1)	−47 (7)	−11 (2)	48 (7)	12 (1)	−42 (4)	−11 (1)

Mean (standard deviation) of maxima (P1, P2, ×10^–3^ m/s), minima (N1, N2, ×10^–3^ m/s) and average rate of change (P1-N1, N1-P2, P2-N2, ×10^–3^ m/s^2^) of normalized frontal-plane *H* of each locomotion condition during four phases

**Fig. 4:**
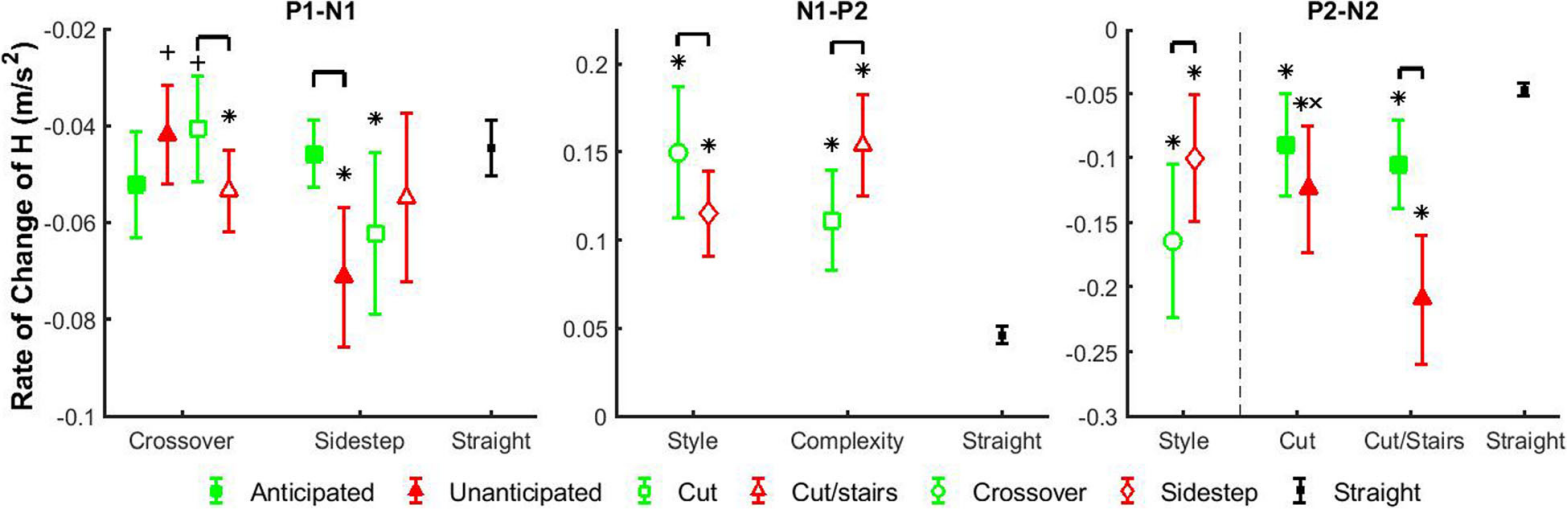
Average and standard deviation of the average rate of change (P1-N1, N1-P2, P2-N2) of frontal-plane whole-body angular momentum (*H*) in different transition phases. Comparisons were performed among anticipation states (filled shapes), cutting styles (unfilled circles and diamonds) and complexity tasks (unfilled squares and triangles). Green filled squares indicate anticipated states, and red filled triangles indicate unanticipated states. Green unfilled squares indicate cuts, and red unfilled triangles indicate cut/stairs transitions. Green unfilled circles indicate crossover styles, and red diamond indicate sidestep styles. Each marker above represents a walking condition of a main factors or an interaction scenario of two main factors, and consists of different levels of interactions or main factor, respectively. Comparisons were also performed between each transition scenario and straight walk. Black dots indicate straight walk in both anticipation states. Brackets indicate significant differences between two transition scenarios. Significant differences between each transition scenario and straight walking are indicated by ‘*’. ‘+’ indicates significant differences between crossover and sidestep cut styles within the same anticipation states, and ‘×’ indicates significant differences between cut and cut/stairs within the same anticipation states or the same cut styles

During the Transition Phase, the average rate of change between P1-N1 had significant anticipation and cutting style main effects, as well as anticipation by cutting style and cutting style by complexity interaction effects. The negative peak N1 had significant anticipation, cutting style, and complexity main effects, as well as anticipation by cutting style, and cutting style by complexity interaction effects. Within unanticipated states, the magnitudes of P1-N1 and N1 increased 70 and 46%, respectively, in sidestep style compared to crossover style (*p* < 0.0001 and *p* = 0.0003), while they are not different within anticipated states (Fig. 3, Fig. 4). For crossover style, the magnitudes of both P1-N1 and N1 increased 32 and 78%, respectively, in more complex cut/stairs transitions relative to cuts (*p* = 0.037 and *p* < 0.0001). However, for sidestep style the magnitudes of P1-N1 and N1 increased 56 and 43%, respectively, in unanticipated states compared to anticipated states (*p* < 0.0001 and *p* = 0.0006).

During the Correction Phase, the average rate of change between N1-P2 had significant main effects of cutting style and complexity. N1-P2 increased 30% in crossover style relative to sidestep style (*p* < 0.0001), and increased 38% in cut/stairs transitions compared to cuts (*p* < 0.0001). All main effects of positive peak P2 were significant, and significant anticipation by complexity and cutting style by complexity interaction effects were also found. Within both anticipated and unanticipated states, P2 increased 41 and 75%, respectively, in cut/stairs transitions relative to cuts (*p* = 0.010 and *p* < 0.0001). For more complex cut/stairs transitions, P2 increased 45% in unanticipated state compared to anticipated state (*p* = 0.0001). P2 increased 66% in cut/stairs transitions relative to cuts for crossover style (*p* < 0.0001), while it was not different for sidestep style. Finally, for both cut and cut/stairs tasks, P2 increased 176 and 227%, respectively, in crossover style compared to sidestep style (*p* < 0.010 and *p* < 0.0001).

During the Completion Phase, all main effects of the average rate of change P2-N2 were significant, and the anticipation by complexity interaction effect was also significant. In general, the magnitude of P2-N2 was 64% larger in crossover transitions relative to sidestep transitions (*p* < 0.0001). For more complex transitions, the magnitude of P2-N2 was 100% greater in unanticipated state compared to anticipated state (*p* < 0.0001). Negative peak N2 had significant anticipation, cutting style, and complexity main effects, as well as anticipation by complexity and cutting style by complexity interaction effects. For both cuts and cut/stairs transitions, the magnitude of N2 increased 91 and 127%, respectively, in unanticipated state relative to anticipated state (*p* < 0.021 and *p* < 0.0001). For sidestep style, the magnitude of N2 was 327% larger in cut/stairs transitions compared to cuts (*p* < 0.0001). In addition, within unanticipated states, both the magnitudes of P2-N2 and N2 increased 69 and 99%, respectively, in cut/stairs transitions relative to cuts (*p* < 0.0001 and *p* < 0.0001).

## Discussion

### Increased task complexity and unanticipated state pose challenges on dynamic balance regulation

We investigated the time-varying frontal-plane dynamic balance based on whole-body angular momentum (*H*) during transitions of altered anticipation, cut style and complexity in healthy young adults. Our first hypothesis was that the peak values of frontal-plane *H* would be larger during unanticipated transitions of increased complexity, such as unanticipated cut/stairs. This hypothesis was supported. During Correction Phase when participants transitioned from level ground to stair-walking (Fig. 1a), positive peak angular momentum P2 was larger in cut/stairs transitions relative to cuts, regardless of anticipation state (Fig. 3). Furthermore, within cut/stairs task, P2 was larger in unanticipated state compared to anticipated state. While it has been shown that increased range of frontal-plane *H* was maintained in steady-state stair ascent compared to straight level walk for healthy subjects [36], our results further suggest that increased *H* was required for unanticipated cut/stairs transitions. The increased peak value of frontal-plane *H* may indicate higher risk of mediolateral fall during unanticipated complex transitions, which is affected by interrupted neuromuscular task planning in unanticipated states [15] and enhanced joint moment requirement for stair walking [19, 20].

To further understand the regulation of *H*, we investigated the average rate of change of *H* that can be considered as the average net external moment about the body’s COM. Our second hypothesis was that the average rate of change of frontal-plane *H* would also be largest during unanticipated transitions of increased complexity. This hypothesis was partially supported. The magnitude of the average rate of change of *H* between N1-P2, during the Correction Phase, was larger in cut/stair-ascent transitions compared to cuts, while there was no effect of anticipation state (Fig. 4). Decreased mediolateral GRFs and vertical moment arms that are main contributors to the mediolateral net external moment likely explain the enhanced slope and range of frontal-plane *H* during early stance of stair ascent relative to level straight walking [36]. Similarly, the increased magnitude of N1-P2 slope indicating larger mediolateral net external moment may explain the increased positive peak P2 during stair-ascent transitions. Furthermore, the gluteus medius has been shown to be the major contributor to maintain frontal-plane angular momentum by rotating the body toward the ipsilateral leg during stance phase [26]. It has been also reported that gluteus medius performed similarly in maintaining mediolateral balance in stair and level walking [37]. Positive angular momentum relates to rotation toward the leading leg, consistent with the functional direction of gluteus medius in maintaining dynamic balance. Thus, the increased frontal-plane *H* may result from reduced gluteus medius activity during a level-stair transition. Previous study also suggested that increased frontal-plane angular momentum during steady-state stair walking may be a necessary strategy to raise body COM while avoiding a trip [36]. Therefore, complex locomotor transitions from level to stair-ascent walking may require a different strategy relative to level transitions, and excessive angular momentum swinging human body toward leading leg may be needed for dynamic balance. These results may be useful for assessing risks of balance-challenged populations during complex locomotor transitions.

### Anticipatory changes in dynamic balance are influenced by cut style

Individuals make cognitive adjustments for their control of dynamic balance before they approach the transition point, and their strategies depend exclusively on cut styles according to our results. During Preparation Phase participants in anticipated states increased the positive peak of *H* (P1) for crossover styles, but reduced the positive peak for sidestep styles compared to straight walking and unanticipated transitions (Fig. 3). Although we did not analyze *H* before P1, style-specific modifications on anticipatory *H* were also performed at the first trailing leg heel strike (Time 0) with the same strategies used for P1 (Fig. 2). These adjustments are understandable because the increased *H* (rotation toward the leading leg) for crossover styles and reduced *H* (rotation away from the leading leg) for sidestep styles were the same as the cut styles. These findings may be a generalized strategy in prepared human walking cuts. A recent study found that during anticipated 90-degree walk turns, angular momentum was not affected by the direction of change as long as individuals use sidestep cut style [38]. Similar conclusions were also made for healthy individuals performing crossover 90-degree cuts to right and left direction [39]. Although these investigations did not compare different cut styles, their results support our findings that anticipatory change of dynamic balance is affected by cut style, but not the direction. Furthermore, these adjustments of whole-body angular momentum may partially result from preparatory control of trunk angular momentum that had the same modification strategy as *H* [40]. Previous study on sidestep cuts also reported that trunk swing is a strategy assisting in moving body COM toward new walking direction [41]. They found that the trunk displaced opposite from the cutting direction before turning, and assisted direction change in an inverted pendulum manner. However, this different trunk strategy was thought to be used in a late cue (unanticipated) transition, and contribute less in an early cue (anticipated) condition. Therefore, individuals in anticipated walking cuts initiated their control of mediolateral dynamic balance in advance to prepare for the direction change, and the swing of upper body segment may contribute to this strategy.

These anticipatory adjustments on *H* may also have implications on the effects of different transition factors on walking dynamic balance regulation. Our results showed that individuals modified their dynamic balance at least one half step before anticipated transitions. This is expected because studies have shown gaze fixation on the future foot landing area before at least two steps [42, 43], modifications on gait parameters for two strides before transition to stairs [24], and high rate of successful direction change when individuals were cued two steps ahead [44]. Nonetheless, subjects adjusted *H* based on cut styles, not task complexity that requires more biomechanical changes. Although it could be argued that individuals prioritize some more closely approaching challenges, they failed to adjust *H* for task complexity at least half step before transitions. This may suggest a priority of cut style over task complexity (cut/stairs) in the “internal model” of human nervous system that regulates locomotion and dynamic balance. This priority may be due to the fear of knee injury during cuts that are associated with increased breaking forces (anterior/posterior GRF) and quadriceps activation [12]. Therefore, healthy adults pre-rotated their body to prepare for incoming cut transitions, which may be an effective strategy to maintain dynamic balance and avoid injuries during these destabilizing tasks. Our findings on the anticipatory adjustments of healthy dynamic balance regulation may provide a baseline to evaluate and improve related routines in rehabilitation training.

### Reactive control of dynamic balance is influenced by cut style

Individuals make reactive changes to dynamic balance in response to unexpected auditory cue of transition, and the strategies are different for each cut style. During sidestep-style transitions, they increased the magnitudes of P1-N1 and N1 for unanticipated states compared to anticipated states, but during crossover styles the magnitudes remain for both anticipation states (Fig. 3, Fig. 4). Furthermore, within unanticipated states the magnitudes of P1-N1 and N1 were greater in sidestep style compared to crossover style, which may be due to the unique mechanisms that individuals used for each cut style. There was a delay of the occurrence of the negative peak N1 during crossover-style transitions compared to straight walking and sidestep-style transitions (Fig. 2). It was at the trailing leg heel-strike for sidestep style transitions and straight walking, but at the subsequent leading leg toe-off for crossover styles. Moreover, *H* in crossover-style transitions was maintained as tightly as straight walking at the trailing leg heel-strike. With limited response time in unanticipated conditions (auditory cue at the initiation of leg swing), participants were not able to change leg swing trajectories rapidly to cross the stance leg, and an unanticipated gait termination was performed in crossover transitions. Although angular momentum was tightly regulated, unanticipated crossover transition with gait termination may still be challenging for balance-impaired populations [23]. Furthermore, as direction change continued participants in unanticipated crossover transitions used the leading leg to turn to the new direction in a sidestep style. However, this is only an initiation of direction change because *H* was still in the direction away from the leading leg, i.e., opposite to the new direction of travel. This mechanism was similar to the previously reported control strategy of body COM in the initiation of walking direction change where the trunk was displaced to the opposite of new direction [41]. However, individuals in unanticipated sidestep transitions may easily swing the trailing leg and trunk [40] away from the leading leg direction with increased negative momentum. Thus, in response to unanticipated walking direction change crossover style may require rapid gait termination and inverted-pendulum-style trunk motion to initialize its direction change, while sidestep may take advantage of the momentum during leg swing to be a more effective maneuver for quicker changing of locomotion direction [12]. Nonetheless, the potential balance challenge in the gait termination of unanticipated crossover transitions and increased momentum in unanticipated sidestep transitions may still need to be carefully considered in rehabilitation training.

### Limitations and future considerations

One limitation of our study is that we did not include arms in the model used to calculate *H*. Although arm swing may contribute to transverse-plane *H* during treadmill walking [45] and anteroposterior fall recovery [46], evidence have shown that the magnitude of contributions to frontal-plane *H* from the arms are very small relative to the trunk and legs during normal walking [8] and 90-degree turn [38]. Our statistical results may also be limited by the modest number of participants. To mitigate this, we collected five trials of each condition for each subject and analyzed the results objectively. While we used subject-averaged data for analysis, the effect sizes turned out to be large (η_p_^2^ > 0.14) for all statistically significant results. Nonetheless, the results should be interpreted as initial findings given the modest number of participants. Another limitation may be that we evaluated *H* in the lab (inertial) frame compared to recent research on dynamic balance in the body moving reference frame [47]. *H* in inertial frame can be directly related to ground reaction force measurements. We also believe that during walking turns, dynamic balance in the direction of inertial frame is more endangered, evidenced by significantly larger peak values compared to *H* in anatomical medial/lateral direction (not published). Finally, *H* in this study was not normalized by walking velocity. Although previous studies showed different angular momenta with walking speed [9, 32], we wanted to incorporate the velocity information in the single metric *H*, which may be part of the strategy that individuals used to maintain dynamic balance during walking transition, as increased speed of transition can adversely influence walking stability of both young and old individuals [48]. Future work is also needed to understand segmental contributions to angular momentum in each transition task so that specific strategy of dynamic balance control in locomotor transitions can be apprehended and targeted for rehabilitation training. Future experiment and analysis on patients walking during locomotor transitions may also be useful to fully understand dynamic balance regulation mechanism and improve rehabilitation training of these populations.

## Conclusions

The results of this study underlined the detrimental effects of unanticipated states and task complexity on dynamic balance during walking transitions. These results imply increased demand of neuromuscular system and functional deficits of individuals with neuromuscular disorders during these tasks. In addition, cutting style influenced frontal plane dynamic balance. These healthy young adults made anticipatory adjustments of dynamic balance before transition based on cutting style, but not complexity (i.e., terrain). Reactive control of dynamic balance in response to unanticipated direction change was also affected by cutting style with specific advantage and challenge to the locomotion tasks.

## Data Availability

The datasets used and/or analyzed during the current study are available from the corresponding author on reasonable request.

## Abbreviations

*H*: Whole-body angular momentum
*M*_*external*_: Net external moment
COM: Center of mass
GRF: Ground reaction force
$\dot{H}$: Time derivative of whole-body angular momentum
SD: Standard deviation
